# Bis[1-(1-adamantyliminomethyl)-2-naphtholato-κ^2^ *N*,*O*]cobalt(II)

**DOI:** 10.1107/S1600536808027475

**Published:** 2008-09-06

**Authors:** Jimmy U. Franco, Marilyn M. Olmstead, Justin C. Hammons

**Affiliations:** a Department of Chemistry, University of California, One Shields Avenue, Davis, CA 95616, USA

## Abstract

The title compound, [Co(C_21_H_22_NO)_2_], crystallizes with two mol­ecules in the asymmetric unit. The coordination environments of the two Co^II^ ions are distorted tetra­hedral. The primary structural difference between the two independent complex mol­ecules lies in the orientations of their adamantyl groups.

## Related literature

For structures of the ligand and a copper complex of the ligand, see: Acevedo-Arauz *et al.* (1992 ▶). For a carboxyl­ato-bridged Rh dimer with axial coordination of the neutral ligand, see: Franco *et al.* (2007 ▶).
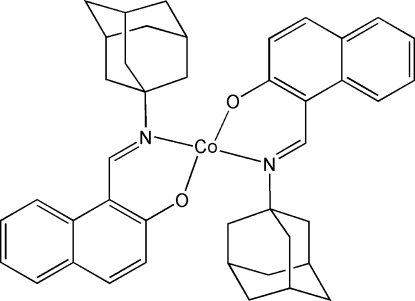


## Experimental

### 

#### Crystal data

[Co(C_21_H_22_NO)_2_]
*M* *_r_* = 667.72
Triclinic, 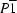

*a* = 13.9055 (5) Å
*b* = 14.3576 (5) Å
*c* = 19.5899 (6) Å
α = 69.938 (2)°
β = 71.219 (2)°
γ = 68.473 (2)°
*V* = 3330.4 (2) Å^3^
*Z* = 4
Mo *K*α radiation
μ = 0.56 mm^−1^
*T* = 90 (2) K
0.33 × 0.24 × 0.18 mm

#### Data collection

Bruker SMART APEXII diffractometer
Absorption correction: multi-scan (*SADABS*; Sheldrick, 1996 ▶) *T*_min_ = 0.876, *T*_max_ = 0.925
42959 measured reflections
15246 independent reflections
11999 reflections with *I* > 2σ(*I*)
*R*_int_ = 0.038

#### Refinement

*R*[*F* ^2^ > 2σ(*F* ^2^)] = 0.036
*wR*(*F* ^2^) = 0.088
*S* = 1.02
15246 reflections
847 parameters
H-atom parameters constrained
Δρ_max_ = 0.39 e Å^−3^
Δρ_min_ = −0.32 e Å^−3^

### 

Data collection: *APEX2* (Bruker, 2007 ▶); cell refinement: *SAINT* (Bruker, 2007 ▶); data reduction: *SAINT*; program(s) used to solve structure: *SHELXS97* (Sheldrick, 2008 ▶); program(s) used to refine structure: *SHELXL97* (Sheldrick, 2008 ▶); molecular graphics: *SHELXTL* (Sheldrick, 2008 ▶); software used to prepare material for publication: *SHELXL97*.

## Supplementary Material

Crystal structure: contains datablocks global, I. DOI: 10.1107/S1600536808027475/gk2163sup1.cif

Structure factors: contains datablocks I. DOI: 10.1107/S1600536808027475/gk2163Isup2.hkl

Additional supplementary materials:  crystallographic information; 3D view; checkCIF report

## Figures and Tables

**Table:** 

Co1—O1	1.9051 (12)
Co1—O2	1.9192 (12)
Co1—N1	1.9962 (14)
Co1—N2	2.0019 (14)
Co2—O3	1.9139 (12)
Co2—O4	1.9194 (12)
Co2—N3	1.9945 (13)
Co2—N4	1.9849 (14)

**Table:** 

O1—Co1—O2	115.23 (5)
O1—Co1—N1	94.93 (5)
O2—Co1—N1	117.15 (5)
O1—Co1—N2	115.95 (5)
O2—Co1—N2	93.16 (5)
N1—Co1—N2	122.20 (6)
O3—Co2—O4	114.40 (5)
O3—Co2—N3	94.94 (5)
O3—Co2—N4	116.97 (5)
O4—Co2—N3	115.20 (5)
O4—Co2—N4	94.63 (5)
N3—Co2—N4	122.25 (6)

**Fig. 1.**
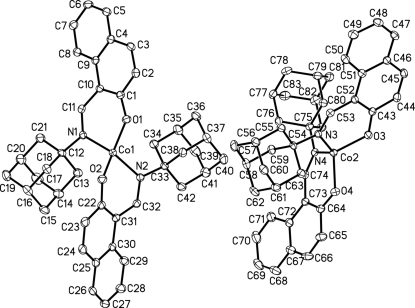
A view of both molecules in the asymmetric unit of the title compound with the atom numbering scheme. Displacement ellipsoids are drawn at the 50% probability level.

## supplementary crystallographic information

### Comment

The title Co^II^ complex has two bidentate Schiff base ligands in a typical coordination mode involving N and O donor atoms. A similar bidentate mode of coordination for the same ligand has been reported in the bis-Cu^II^ complex (Acevedo-Arauz *et al.*, 1992). Earlier (Franco *et al.*, 2007), we reported the structure of a carboxylato-bridged Rh dimer in which the neutral ligand binds axially in a monodentate mode through the oxygen atom and forms an intramolecular hydrogen bond to the N atom. The bidentate coordination mode of the title complex is fundamentally different because the ligand has been deprotonated.

There are two molecules in the asymmetric unit. These are depicted in Fig. 1 as they occur in the structure. The coordination geometry for Co^II^ is in the normal range for complexes of this type. The N_2_O_2_ donor set forms a distorted tetrahedral coordination with angles at Co ranging from 93.16 (5)° to 122.20 (6)° in the complex involving Co1 and 94.63 (5)° to 122.25 (6)° in the complex involving Co2. A calculation of the least-squares plane of the thirteen atoms comprising the 2-naphthol-iminomethyl ligand reveals a marked deviation from planarity at the imino carbon atom. An inspection of the structure indicates that this buckling is likely due to a short intramolecular contact between the C—H hydrogen and one of the naphthol H atoms. The deviations of this carbon from the four different ligand planes are 0.1146 (13) Å, C11; 0.1861 (14) Å, C32; 0.0992 (14) Å, C53; 0.1223 (14) Å, C74.

In addition to these differences in buckling of the Schiff base, the two molecules differ in the rotation of the adamantyl substituent. These are attributable to packing interactions.

### Experimental

The ligand, 1-(1-adamantyl)iminomethyl)-2-naphthol, was prepared as previously described (Franco *et al.*, 2007). The Co complex was prepared as follows. In a 100 ml round bottom flask, 50 mg of 1-(1-adamantyl)iminomethyl)-2-naphthol (16 mmol) was dissolved in 30 ml of methanol, followed by addition of 7 mg of methanolic NaOH (16 mmol). After the solution was stirred for ten minutes, 16 mg of CoCl_2_^.^2(H_2_O) (8 mmol) dissolved in 15 ml of methanol was added and the reaction mixture stirred for another three hours at room temperature. The solution was then filtered to remove any solids and the solvent removed by use of a rotary evaporator. The residue was crystallized by slow evaporation of a methanol solution to afford red-orange crystals, yield 84%.

### Refinement

The C-bound H atoms were positioned geometrically with C—H = 0.95–0.99 Å, and allowed to ride on their parent atoms with *U*_iso_(H) = 1.2 *U*_eq_(C).

### Crystal data

**Table:**

[Co(C_21_H_22_NO)_2_]	*Z* = 4
*M**_r_* = 667.72	*F*(000) = 1412
Triclinic, *P*1	*D*_x_ = 1.332 Mg m^−^^3^
Hall symbol: -P 1	Mo *K*α radiation, λ = 0.71073 Å
*a* = 13.9055 (5) Å	Cell parameters from 5583 reflections
*b* = 14.3576 (5) Å	θ = 2.8–27.4°
*c* = 19.5899 (6) Å	µ = 0.56 mm^−^^1^
α = 69.938 (2)°	*T* = 90 K
β = 71.219 (2)°	Plate, red-orange
γ = 68.473 (2)°	0.33 × 0.24 × 0.18 mm
*V* = 3330.4 (2) Å^3^	

### Data collection

**Table:**

Bruker SMART APEXII diffractometer	15246 independent reflections
Radiation source: fine-focus sealed tube	11999 reflections with *I* > 2σ(*I*)
graphite	*R*_int_ = 0.038
Detector resolution: 8.3 pixels mm^-1^	θ_max_ = 27.5°, θ_min_ = 2.8°
ω scans	*h* = −18→18
Absorption correction: multi-scan (SADABS; Sheldrick, 1996)	*k* = −18→18
*T*_min_ = 0.876, *T*_max_ = 0.925	*l* = −25→25
42959 measured reflections	

### Refinement

**Table:**

Refinement on *F*^2^	Primary atom site location: structure-invariant direct methods
Least-squares matrix: full	Secondary atom site location: difference Fourier map
*R*[*F*^2^ > 2σ(*F*^2^)] = 0.036	Hydrogen site location: inferred from neighbouring sites
*wR*(*F*^2^) = 0.088	H-atom parameters constrained
*S* = 1.02	*w* = 1/[σ^2^(*F*_o_^2^) + (0.0384*P*)^2^ + 1.0354*P*] where *P* = (*F*_o_^2^ + 2*F*_c_^2^)/3
15246 reflections	(Δ/σ)_max_ = 0.002
847 parameters	Δρ_max_ = 0.39 e Å^−^^3^
0 restraints	Δρ_min_ = −0.32 e Å^−^^3^

### Special details

**Table:**

Geometry. All e.s.d.'s (except the e.s.d. in the dihedral angle between two l.s. planes) are estimated using the full covariance matrix. The cell e.s.d.'s are taken into account individually in the estimation of e.s.d.'s in distances, angles and torsion angles; correlations between e.s.d.'s in cell parameters are only used when they are defined by crystal symmetry. An approximate (isotropic) treatment of cell e.s.d.'s is used for estimating e.s.d.'s involving l.s. planes.
Refinement. Refinement of *F*^2^ against ALL reflections. The weighted *R*-factor *wR* and goodness of fit *S* are based on *F*^2^, conventional *R*-factors *R* are based on *F*, with *F* set to zero for negative *F*^2^. The threshold expression of *F*^2^ > σ(*F*^2^) is used only for calculating *R*-factors(gt) *etc*. and is not relevant to the choice of reflections for refinement. *R*-factors based on *F*^2^ are statistically about twice as large as those based on *F*, and *R*- factors based on ALL data will be even larger.

### Fractional atomic coordinates and isotropic or equivalent isotropic displacement parameters (Å^2^)

**Table:**

	*x*	*y*	*z*	*U*_iso_*/*U*_eq_	
Co1	0.588500 (17)	0.756438 (17)	0.086566 (12)	0.01448 (6)	
O2	0.44739 (9)	0.80539 (9)	0.14274 (7)	0.0199 (3)	
O1	0.69699 (9)	0.78062 (9)	0.11194 (6)	0.0191 (3)	
N1	0.62251 (11)	0.83082 (10)	−0.02092 (8)	0.0142 (3)	
N2	0.58369 (11)	0.60961 (11)	0.11945 (8)	0.0148 (3)	
C1	0.77555 (13)	0.81402 (13)	0.06543 (9)	0.0165 (3)	
C2	0.85436 (14)	0.81505 (14)	0.09778 (10)	0.0200 (4)	
H2	0.8472	0.7904	0.1503	0.024*	
C3	0.93834 (14)	0.85025 (13)	0.05544 (10)	0.0196 (4)	
H3	0.9875	0.8518	0.0790	0.024*	
C4	0.95482 (13)	0.88506 (13)	−0.02376 (10)	0.0173 (3)	
C5	1.04467 (14)	0.91845 (13)	−0.06732 (10)	0.0205 (4)	
H5	1.0930	0.9203	−0.0432	0.025*	
C6	1.06366 (14)	0.94821 (14)	−0.14352 (11)	0.0222 (4)	
H6	1.1239	0.9715	−0.1721	0.027*	
C7	0.99276 (14)	0.94382 (13)	−0.17891 (10)	0.0203 (4)	
H7	1.0059	0.9632	−0.2318	0.024*	
C8	0.90448 (13)	0.91185 (13)	−0.13780 (10)	0.0175 (3)	
H8	0.8583	0.9088	−0.1631	0.021*	
C9	0.88031 (13)	0.88328 (12)	−0.05871 (9)	0.0154 (3)	
C10	0.78698 (13)	0.85024 (12)	−0.01287 (9)	0.0150 (3)	
C11	0.70689 (13)	0.86179 (12)	−0.04951 (9)	0.0149 (3)	
H11	0.7173	0.8965	−0.1011	0.018*	
C12	0.54877 (13)	0.85018 (13)	−0.06884 (9)	0.0155 (3)	
C13	0.54044 (14)	0.74344 (13)	−0.06166 (10)	0.0201 (4)	
H13A	0.6107	0.6999	−0.0824	0.024*	
H13B	0.5188	0.7089	−0.0083	0.024*	
C14	0.45935 (15)	0.75395 (15)	−0.10333 (11)	0.0246 (4)	
H14	0.4550	0.6838	−0.0981	0.029*	
C15	0.35098 (15)	0.81967 (16)	−0.06975 (11)	0.0269 (4)	
H15A	0.3291	0.7860	−0.0162	0.032*	
H15B	0.2973	0.8257	−0.0954	0.032*	
C16	0.35748 (14)	0.92782 (15)	−0.07856 (10)	0.0246 (4)	
H16	0.2864	0.9711	−0.0572	0.029*	
C17	0.49498 (16)	0.80561 (16)	−0.18567 (11)	0.0298 (5)	
H17A	0.4440	0.8117	−0.2135	0.036*	
H17B	0.5654	0.7628	−0.2068	0.036*	
C18	0.43936 (13)	0.91824 (14)	−0.03720 (10)	0.0199 (4)	
H18A	0.4441	0.9879	−0.0432	0.024*	
H18B	0.4165	0.8870	0.0167	0.024*	
C19	0.39209 (15)	0.97930 (15)	−0.16090 (11)	0.0276 (4)	
H19A	0.3957	1.0492	−0.1666	0.033*	
H19B	0.3396	0.9868	−0.1881	0.033*	
C20	0.50118 (15)	0.91321 (16)	−0.19370 (10)	0.0262 (4)	
H20	0.5238	0.9472	−0.2477	0.031*	
C21	0.58291 (14)	0.90275 (15)	−0.15148 (10)	0.0215 (4)	
H21A	0.6538	0.8611	−0.1730	0.026*	
H21B	0.5876	0.9721	−0.1568	0.026*	
C22	0.37224 (13)	0.76579 (13)	0.15094 (9)	0.0175 (4)	
C23	0.26509 (14)	0.82850 (14)	0.17133 (11)	0.0239 (4)	
H23	0.2530	0.8945	0.1784	0.029*	
C24	0.18098 (14)	0.79536 (14)	0.18072 (11)	0.0244 (4)	
H24	0.1113	0.8384	0.1948	0.029*	
C25	0.19442 (14)	0.69776 (14)	0.17003 (10)	0.0207 (4)	
C26	0.10572 (14)	0.66529 (15)	0.17852 (10)	0.0230 (4)	
H26	0.0363	0.7089	0.1924	0.028*	
C27	0.11792 (15)	0.57242 (15)	0.16719 (10)	0.0257 (4)	
H27	0.0576	0.5514	0.1735	0.031*	
C28	0.22060 (15)	0.50839 (15)	0.14610 (10)	0.0243 (4)	
H28	0.2296	0.4440	0.1377	0.029*	
C29	0.30838 (14)	0.53796 (14)	0.13740 (10)	0.0209 (4)	
H29	0.3770	0.4938	0.1224	0.025*	
C30	0.29912 (13)	0.63255 (13)	0.15019 (9)	0.0178 (4)	
C31	0.38928 (13)	0.66629 (13)	0.14309 (9)	0.0171 (3)	
C32	0.49285 (13)	0.59156 (13)	0.13748 (9)	0.0161 (3)	
H32	0.4946	0.5213	0.1482	0.019*	
C33	0.68321 (13)	0.52224 (13)	0.12298 (9)	0.0154 (3)	
C34	0.76264 (13)	0.54495 (14)	0.04841 (9)	0.0189 (4)	
H34A	0.7351	0.5455	0.0075	0.023*	
H34B	0.7713	0.6141	0.0386	0.023*	
C35	0.87028 (14)	0.46254 (14)	0.05038 (10)	0.0232 (4)	
H35	0.9212	0.4782	0.0015	0.028*	
C36	0.91327 (14)	0.46301 (15)	0.11315 (10)	0.0231 (4)	
H36A	0.9832	0.4105	0.1144	0.028*	
H36B	0.9225	0.5317	0.1041	0.028*	
C37	0.83511 (14)	0.43872 (14)	0.18776 (10)	0.0220 (4)	
H37	0.8632	0.4387	0.2288	0.026*	
C38	0.72696 (13)	0.52142 (13)	0.18607 (10)	0.0182 (4)	
H38A	0.6766	0.5062	0.2345	0.022*	
H38B	0.7351	0.5904	0.1779	0.022*	
C39	0.85710 (15)	0.35565 (15)	0.06471 (11)	0.0285 (4)	
H39A	0.8307	0.3543	0.0239	0.034*	
H39B	0.9265	0.3022	0.0661	0.034*	
C40	0.82098 (15)	0.33239 (14)	0.20207 (11)	0.0269 (4)	
H40A	0.7706	0.3168	0.2504	0.032*	
H40B	0.8899	0.2784	0.2044	0.032*	
C41	0.77850 (15)	0.33235 (14)	0.13903 (12)	0.0264 (4)	
H41A	0.7695	0.2628	0.1482	0.032*	
C42	0.67056 (14)	0.41526 (13)	0.13678 (11)	0.0225 (4)	
H42A	0.6424	0.4149	0.0965	0.027*	
H42B	0.6195	0.3995	0.1847	0.027*	
Co2	0.886744 (17)	0.185711 (17)	0.585004 (12)	0.01390 (6)	
O3	1.00067 (9)	0.10345 (9)	0.63422 (6)	0.0172 (2)	
O4	0.76314 (9)	0.13745 (9)	0.62356 (6)	0.0182 (3)	
N3	0.87112 (10)	0.31587 (10)	0.60847 (7)	0.0139 (3)	
N4	0.90708 (11)	0.17854 (10)	0.48169 (8)	0.0143 (3)	
C43	1.06441 (13)	0.14134 (13)	0.64622 (9)	0.0149 (3)	
C44	1.16275 (13)	0.06899 (13)	0.66230 (9)	0.0183 (4)	
H44	1.1779	−0.0011	0.6623	0.022*	
C45	1.23434 (13)	0.09768 (13)	0.67740 (9)	0.0187 (4)	
H45	1.2983	0.0472	0.6881	0.022*	
C46	1.21613 (13)	0.20208 (13)	0.67764 (9)	0.0158 (3)	
C47	1.29146 (13)	0.23070 (14)	0.69404 (9)	0.0191 (4)	
H47	1.3538	0.1791	0.7067	0.023*	
C48	1.27664 (14)	0.33105 (14)	0.69216 (10)	0.0214 (4)	
H48	1.3280	0.3493	0.7033	0.026*	
C49	1.18440 (14)	0.40625 (14)	0.67358 (10)	0.0220 (4)	
H49	1.1737	0.4763	0.6717	0.026*	
C50	1.10900 (14)	0.38079 (14)	0.65797 (10)	0.0200 (4)	
H50	1.0473	0.4338	0.6455	0.024*	
C51	1.12092 (13)	0.27747 (13)	0.66006 (9)	0.0158 (3)	
C52	1.04294 (13)	0.24605 (13)	0.64497 (9)	0.0153 (3)	
C53	0.94385 (13)	0.32323 (13)	0.63285 (9)	0.0153 (3)	
H53	0.9304	0.3868	0.6441	0.018*	
C54	0.77189 (13)	0.40338 (12)	0.60191 (9)	0.0143 (3)	
C55	0.77324 (13)	0.50404 (13)	0.61211 (10)	0.0176 (4)	
H55A	0.8321	0.5282	0.5744	0.021*	
H55B	0.7850	0.4913	0.6622	0.021*	
C56	0.66674 (14)	0.58790 (13)	0.60358 (10)	0.0198 (4)	
H56	0.6685	0.6532	0.6102	0.024*	
C57	0.64941 (15)	0.60857 (14)	0.52579 (11)	0.0241 (4)	
H57A	0.7076	0.6330	0.4873	0.029*	
H57B	0.5817	0.6630	0.5199	0.029*	
C58	0.64665 (14)	0.50811 (14)	0.51598 (10)	0.0218 (4)	
H58	0.6350	0.5210	0.4653	0.026*	
C59	0.75287 (13)	0.42466 (13)	0.52432 (9)	0.0177 (4)	
H59A	0.7520	0.3600	0.5171	0.021*	
H59B	0.8114	0.4482	0.4856	0.021*	
C60	0.57627 (14)	0.55089 (14)	0.66338 (10)	0.0216 (4)	
H60A	0.5871	0.5387	0.7136	0.026*	
H60B	0.5077	0.6046	0.6586	0.026*	
C61	0.57431 (14)	0.45053 (14)	0.65338 (10)	0.0208 (4)	
H61	0.5153	0.4261	0.6922	0.025*	
C62	0.55641 (14)	0.47087 (14)	0.57561 (11)	0.0230 (4)	
H62A	0.5539	0.4065	0.5690	0.028*	
H62B	0.4878	0.5241	0.5703	0.028*	
C63	0.68031 (13)	0.36759 (13)	0.66175 (9)	0.0171 (3)	
H63A	0.6786	0.3020	0.6566	0.020*	
H63B	0.6917	0.3546	0.7119	0.020*	
C64	0.70488 (13)	0.13519 (12)	0.58424 (10)	0.0175 (4)	
C65	0.60514 (14)	0.11461 (14)	0.62443 (11)	0.0233 (4)	
H65	0.5848	0.1052	0.6770	0.028*	
C66	0.53954 (14)	0.10844 (14)	0.58876 (12)	0.0261 (4)	
H66	0.4754	0.0923	0.6173	0.031*	
C67	0.56351 (14)	0.12523 (13)	0.51025 (11)	0.0221 (4)	
C68	0.49154 (15)	0.12397 (14)	0.47355 (13)	0.0288 (5)	
H68	0.4273	0.1084	0.5025	0.035*	
C69	0.51192 (16)	0.14445 (15)	0.39802 (13)	0.0318 (5)	
H69	0.4632	0.1422	0.3744	0.038*	
C70	0.60583 (17)	0.16889 (15)	0.35577 (12)	0.0311 (5)	
H70	0.6201	0.1847	0.3029	0.037*	
C71	0.67797 (15)	0.17039 (14)	0.38951 (11)	0.0246 (4)	
H71	0.7411	0.1872	0.3593	0.029*	
C72	0.66044 (14)	0.14760 (13)	0.46771 (10)	0.0190 (4)	
C73	0.73407 (13)	0.14876 (12)	0.50606 (10)	0.0165 (3)	
C74	0.83515 (13)	0.16210 (12)	0.46196 (9)	0.0158 (3)	
H74	0.8516	0.1584	0.4118	0.019*	
C75	1.00216 (13)	0.19701 (13)	0.42137 (9)	0.0142 (3)	
C76	0.96679 (13)	0.29700 (13)	0.36171 (9)	0.0169 (3)	
H76A	0.9173	0.2890	0.3388	0.020*	
H76B	0.9290	0.3555	0.3853	0.020*	
C77	1.06352 (14)	0.32036 (13)	0.30118 (10)	0.0192 (4)	
H77	1.0397	0.3849	0.2624	0.023*	
C78	1.13780 (14)	0.33519 (14)	0.33722 (10)	0.0213 (4)	
H78A	1.1002	0.3939	0.3606	0.026*	
H78B	1.2000	0.3514	0.2986	0.026*	
C79	1.17465 (14)	0.23610 (14)	0.39641 (10)	0.0208 (4)	
H79	1.2231	0.2458	0.4199	0.025*	
C80	1.07802 (13)	0.21172 (14)	0.45621 (10)	0.0184 (4)	
H80A	1.1018	0.1479	0.4946	0.022*	
H80B	1.0404	0.2691	0.4808	0.022*	
C81	1.23330 (14)	0.14586 (14)	0.35980 (11)	0.0239 (4)	
H81A	1.2581	0.0818	0.3977	0.029*	
H81B	1.2961	0.1606	0.3212	0.029*	
C82	1.15870 (14)	0.13049 (14)	0.32418 (10)	0.0220 (4)	
H82	1.1972	0.0716	0.3003	0.026*	
C83	1.12133 (14)	0.22924 (14)	0.26513 (10)	0.0222 (4)	
H83A	1.0729	0.2195	0.2422	0.027*	
H83B	1.1831	0.2441	0.2254	0.027*	
C84	1.06196 (13)	0.10605 (13)	0.38499 (10)	0.0188 (4)	
H84A	1.0141	0.0946	0.3626	0.023*	
H84B	1.0858	0.0421	0.4233	0.023*	

### Atomic displacement parameters (Å^2^)

**Table:**

	*U*^11^	*U*^22^	*U*^33^	*U*^12^	*U*^13^	*U*^23^
Co1	0.01407 (12)	0.01428 (12)	0.01469 (12)	−0.00514 (9)	−0.00332 (9)	−0.00202 (9)
O2	0.0169 (6)	0.0188 (6)	0.0221 (6)	−0.0056 (5)	−0.0016 (5)	−0.0049 (5)
O1	0.0197 (6)	0.0236 (7)	0.0159 (6)	−0.0113 (5)	−0.0040 (5)	−0.0015 (5)
N1	0.0145 (7)	0.0129 (7)	0.0154 (7)	−0.0026 (5)	−0.0047 (6)	−0.0039 (6)
N2	0.0136 (7)	0.0157 (7)	0.0139 (7)	−0.0037 (6)	−0.0023 (5)	−0.0035 (6)
C1	0.0155 (8)	0.0152 (8)	0.0189 (9)	−0.0049 (7)	−0.0038 (7)	−0.0039 (7)
C2	0.0212 (9)	0.0211 (9)	0.0170 (9)	−0.0057 (7)	−0.0075 (7)	−0.0016 (7)
C3	0.0177 (9)	0.0190 (9)	0.0252 (9)	−0.0053 (7)	−0.0103 (7)	−0.0045 (7)
C4	0.0162 (8)	0.0127 (8)	0.0225 (9)	−0.0028 (7)	−0.0052 (7)	−0.0048 (7)
C5	0.0167 (9)	0.0176 (9)	0.0287 (10)	−0.0047 (7)	−0.0073 (7)	−0.0060 (8)
C6	0.0149 (9)	0.0194 (9)	0.0297 (10)	−0.0066 (7)	−0.0007 (7)	−0.0053 (8)
C7	0.0203 (9)	0.0163 (9)	0.0209 (9)	−0.0044 (7)	−0.0014 (7)	−0.0048 (7)
C8	0.0167 (8)	0.0141 (8)	0.0217 (9)	−0.0027 (7)	−0.0050 (7)	−0.0059 (7)
C9	0.0157 (8)	0.0092 (8)	0.0196 (9)	−0.0020 (6)	−0.0035 (7)	−0.0039 (7)
C10	0.0140 (8)	0.0130 (8)	0.0188 (9)	−0.0038 (6)	−0.0040 (7)	−0.0049 (7)
C11	0.0171 (8)	0.0126 (8)	0.0142 (8)	−0.0039 (7)	−0.0035 (7)	−0.0029 (6)
C12	0.0155 (8)	0.0185 (9)	0.0153 (8)	−0.0061 (7)	−0.0048 (7)	−0.0049 (7)
C13	0.0206 (9)	0.0191 (9)	0.0239 (9)	−0.0061 (7)	−0.0080 (7)	−0.0065 (7)
C14	0.0279 (10)	0.0247 (10)	0.0294 (10)	−0.0114 (8)	−0.0112 (8)	−0.0083 (8)
C15	0.0231 (10)	0.0374 (11)	0.0261 (10)	−0.0141 (9)	−0.0117 (8)	−0.0036 (9)
C16	0.0176 (9)	0.0303 (10)	0.0261 (10)	−0.0027 (8)	−0.0090 (8)	−0.0083 (8)
C17	0.0322 (11)	0.0407 (12)	0.0248 (10)	−0.0101 (9)	−0.0107 (9)	−0.0146 (9)
C18	0.0188 (9)	0.0209 (9)	0.0205 (9)	−0.0028 (7)	−0.0072 (7)	−0.0065 (7)
C19	0.0290 (11)	0.0281 (10)	0.0280 (10)	−0.0064 (8)	−0.0170 (9)	−0.0020 (8)
C20	0.0282 (10)	0.0362 (11)	0.0167 (9)	−0.0130 (9)	−0.0084 (8)	−0.0027 (8)
C21	0.0219 (9)	0.0270 (10)	0.0174 (9)	−0.0101 (8)	−0.0059 (7)	−0.0032 (8)
C22	0.0168 (8)	0.0173 (9)	0.0144 (8)	−0.0049 (7)	−0.0022 (7)	−0.0005 (7)
C23	0.0202 (9)	0.0164 (9)	0.0284 (10)	−0.0019 (7)	−0.0036 (8)	−0.0030 (8)
C24	0.0150 (9)	0.0210 (9)	0.0266 (10)	0.0005 (7)	−0.0027 (7)	−0.0013 (8)
C25	0.0171 (9)	0.0230 (9)	0.0163 (9)	−0.0047 (7)	−0.0045 (7)	0.0015 (7)
C26	0.0134 (8)	0.0286 (10)	0.0195 (9)	−0.0051 (7)	−0.0050 (7)	0.0030 (8)
C27	0.0202 (9)	0.0336 (11)	0.0231 (10)	−0.0138 (8)	−0.0078 (8)	0.0021 (8)
C28	0.0250 (10)	0.0288 (10)	0.0225 (10)	−0.0128 (8)	−0.0069 (8)	−0.0038 (8)
C29	0.0174 (9)	0.0255 (10)	0.0188 (9)	−0.0060 (7)	−0.0033 (7)	−0.0054 (8)
C30	0.0159 (8)	0.0197 (9)	0.0133 (8)	−0.0052 (7)	−0.0035 (7)	0.0015 (7)
C31	0.0154 (8)	0.0188 (9)	0.0144 (8)	−0.0055 (7)	−0.0021 (7)	−0.0015 (7)
C32	0.0177 (8)	0.0162 (8)	0.0140 (8)	−0.0061 (7)	−0.0031 (7)	−0.0026 (7)
C33	0.0133 (8)	0.0155 (8)	0.0173 (8)	−0.0031 (7)	−0.0041 (7)	−0.0045 (7)
C34	0.0168 (9)	0.0221 (9)	0.0158 (9)	−0.0056 (7)	−0.0020 (7)	−0.0041 (7)
C35	0.0159 (9)	0.0270 (10)	0.0217 (9)	−0.0012 (7)	−0.0008 (7)	−0.0087 (8)
C36	0.0147 (9)	0.0229 (10)	0.0304 (10)	−0.0026 (7)	−0.0058 (8)	−0.0073 (8)
C37	0.0190 (9)	0.0237 (9)	0.0223 (9)	−0.0017 (7)	−0.0094 (7)	−0.0050 (8)
C38	0.0183 (9)	0.0175 (9)	0.0158 (8)	−0.0028 (7)	−0.0035 (7)	−0.0035 (7)
C39	0.0215 (10)	0.0285 (11)	0.0360 (11)	0.0034 (8)	−0.0083 (8)	−0.0186 (9)
C40	0.0205 (10)	0.0210 (10)	0.0304 (11)	−0.0006 (8)	−0.0071 (8)	−0.0005 (8)
C41	0.0215 (10)	0.0144 (9)	0.0433 (12)	−0.0022 (7)	−0.0090 (9)	−0.0089 (8)
C42	0.0165 (9)	0.0170 (9)	0.0343 (11)	−0.0041 (7)	−0.0063 (8)	−0.0070 (8)
Co2	0.01420 (12)	0.01327 (11)	0.01472 (12)	−0.00393 (9)	−0.00410 (9)	−0.00341 (9)
O3	0.0180 (6)	0.0151 (6)	0.0198 (6)	−0.0049 (5)	−0.0071 (5)	−0.0031 (5)
O4	0.0189 (6)	0.0195 (6)	0.0180 (6)	−0.0086 (5)	−0.0022 (5)	−0.0053 (5)
N3	0.0130 (7)	0.0136 (7)	0.0137 (7)	−0.0028 (5)	−0.0031 (5)	−0.0029 (5)
N4	0.0152 (7)	0.0113 (7)	0.0157 (7)	−0.0040 (5)	−0.0037 (6)	−0.0020 (5)
C43	0.0161 (8)	0.0180 (8)	0.0097 (8)	−0.0059 (7)	−0.0025 (6)	−0.0016 (6)
C44	0.0190 (9)	0.0149 (8)	0.0176 (9)	−0.0025 (7)	−0.0040 (7)	−0.0028 (7)
C45	0.0144 (8)	0.0188 (9)	0.0175 (9)	−0.0007 (7)	−0.0041 (7)	−0.0021 (7)
C46	0.0143 (8)	0.0193 (9)	0.0117 (8)	−0.0047 (7)	−0.0016 (6)	−0.0027 (7)
C47	0.0126 (8)	0.0260 (10)	0.0165 (9)	−0.0022 (7)	−0.0030 (7)	−0.0068 (7)
C48	0.0185 (9)	0.0289 (10)	0.0208 (9)	−0.0090 (8)	−0.0055 (7)	−0.0077 (8)
C49	0.0225 (9)	0.0207 (9)	0.0269 (10)	−0.0067 (8)	−0.0058 (8)	−0.0102 (8)
C50	0.0165 (9)	0.0201 (9)	0.0232 (9)	−0.0012 (7)	−0.0063 (7)	−0.0080 (7)
C51	0.0151 (8)	0.0198 (9)	0.0125 (8)	−0.0048 (7)	−0.0025 (6)	−0.0051 (7)
C52	0.0137 (8)	0.0158 (8)	0.0143 (8)	−0.0029 (7)	−0.0031 (6)	−0.0026 (7)
C53	0.0162 (8)	0.0149 (8)	0.0145 (8)	−0.0045 (7)	−0.0022 (6)	−0.0047 (7)
C54	0.0126 (8)	0.0134 (8)	0.0165 (8)	−0.0006 (6)	−0.0054 (6)	−0.0048 (7)
C55	0.0153 (8)	0.0161 (8)	0.0234 (9)	−0.0036 (7)	−0.0061 (7)	−0.0067 (7)
C56	0.0177 (9)	0.0149 (9)	0.0280 (10)	−0.0018 (7)	−0.0077 (7)	−0.0075 (7)
C57	0.0231 (10)	0.0163 (9)	0.0281 (10)	0.0011 (7)	−0.0107 (8)	−0.0028 (8)
C58	0.0221 (9)	0.0209 (9)	0.0211 (9)	0.0007 (7)	−0.0118 (8)	−0.0051 (7)
C59	0.0187 (9)	0.0177 (9)	0.0170 (8)	−0.0029 (7)	−0.0063 (7)	−0.0051 (7)
C60	0.0167 (9)	0.0196 (9)	0.0270 (10)	0.0014 (7)	−0.0061 (7)	−0.0104 (8)
C61	0.0146 (8)	0.0211 (9)	0.0259 (10)	−0.0040 (7)	−0.0021 (7)	−0.0085 (8)
C62	0.0156 (9)	0.0231 (10)	0.0343 (11)	−0.0005 (7)	−0.0122 (8)	−0.0114 (8)
C63	0.0156 (8)	0.0166 (8)	0.0182 (9)	−0.0029 (7)	−0.0029 (7)	−0.0064 (7)
C64	0.0159 (8)	0.0107 (8)	0.0258 (9)	−0.0031 (7)	−0.0038 (7)	−0.0061 (7)
C65	0.0195 (9)	0.0232 (10)	0.0275 (10)	−0.0086 (8)	0.0023 (8)	−0.0114 (8)
C66	0.0142 (9)	0.0229 (10)	0.0422 (12)	−0.0073 (7)	0.0022 (8)	−0.0151 (9)
C67	0.0162 (9)	0.0146 (9)	0.0381 (11)	−0.0023 (7)	−0.0078 (8)	−0.0105 (8)
C68	0.0166 (9)	0.0195 (10)	0.0558 (14)	−0.0020 (8)	−0.0125 (9)	−0.0155 (9)
C69	0.0283 (11)	0.0212 (10)	0.0568 (14)	−0.0014 (8)	−0.0279 (10)	−0.0124 (10)
C70	0.0367 (12)	0.0248 (10)	0.0373 (12)	−0.0044 (9)	−0.0233 (10)	−0.0055 (9)
C71	0.0263 (10)	0.0191 (9)	0.0317 (11)	−0.0058 (8)	−0.0139 (8)	−0.0043 (8)
C72	0.0185 (9)	0.0111 (8)	0.0286 (10)	−0.0017 (7)	−0.0089 (7)	−0.0060 (7)
C73	0.0157 (8)	0.0114 (8)	0.0232 (9)	−0.0040 (7)	−0.0059 (7)	−0.0039 (7)
C74	0.0188 (8)	0.0124 (8)	0.0155 (8)	−0.0032 (7)	−0.0053 (7)	−0.0027 (7)
C75	0.0140 (8)	0.0143 (8)	0.0141 (8)	−0.0046 (6)	−0.0019 (6)	−0.0040 (6)
C76	0.0169 (8)	0.0141 (8)	0.0190 (9)	−0.0027 (7)	−0.0062 (7)	−0.0033 (7)
C77	0.0222 (9)	0.0160 (9)	0.0169 (9)	−0.0071 (7)	−0.0038 (7)	0.0001 (7)
C78	0.0221 (9)	0.0197 (9)	0.0231 (9)	−0.0119 (8)	0.0011 (7)	−0.0062 (7)
C79	0.0175 (9)	0.0255 (10)	0.0211 (9)	−0.0104 (7)	−0.0037 (7)	−0.0042 (8)
C80	0.0171 (9)	0.0202 (9)	0.0181 (9)	−0.0068 (7)	−0.0042 (7)	−0.0034 (7)
C81	0.0169 (9)	0.0223 (10)	0.0251 (10)	−0.0050 (7)	−0.0002 (7)	−0.0020 (8)
C82	0.0200 (9)	0.0186 (9)	0.0245 (10)	−0.0047 (7)	0.0027 (7)	−0.0101 (8)
C83	0.0214 (9)	0.0283 (10)	0.0173 (9)	−0.0100 (8)	0.0020 (7)	−0.0091 (8)
C84	0.0175 (9)	0.0153 (8)	0.0231 (9)	−0.0049 (7)	−0.0018 (7)	−0.0066 (7)

### Geometric parameters (Å, °)

**Table:**

Co1—O1	1.9051 (12)	Co2—O3	1.9139 (12)
Co1—O2	1.9192 (12)	Co2—O4	1.9194 (12)
Co1—N1	1.9962 (14)	Co2—N3	1.9945 (13)
Co1—N2	2.0019 (14)	Co2—N4	1.9849 (14)
O2—C22	1.306 (2)	O3—C43	1.3088 (19)
O1—C1	1.303 (2)	O4—C64	1.300 (2)
N1—C11	1.295 (2)	N3—C53	1.299 (2)
N1—C12	1.497 (2)	N3—C54	1.493 (2)
N2—C32	1.295 (2)	N4—C74	1.300 (2)
N2—C33	1.491 (2)	N4—C75	1.500 (2)
C1—C10	1.416 (2)	C43—C52	1.414 (2)
C1—C2	1.438 (2)	C43—C44	1.435 (2)
C2—C3	1.352 (2)	C44—C45	1.350 (2)
C2—H2	0.9500	C44—H44	0.9500
C3—C4	1.426 (2)	C45—C46	1.427 (2)
C3—H3	0.9500	C45—H45	0.9500
C4—C5	1.411 (2)	C46—C47	1.412 (2)
C4—C9	1.425 (2)	C46—C51	1.425 (2)
C5—C6	1.368 (3)	C47—C48	1.368 (3)
C5—H5	0.9500	C47—H47	0.9500
C6—C7	1.404 (2)	C48—C49	1.399 (3)
C6—H6	0.9500	C48—H48	0.9500
C7—C8	1.375 (2)	C49—C50	1.375 (2)
C7—H7	0.9500	C49—H49	0.9500
C8—C9	1.418 (2)	C50—C51	1.418 (2)
C8—H8	0.9500	C50—H50	0.9500
C9—C10	1.458 (2)	C51—C52	1.457 (2)
C10—C11	1.442 (2)	C52—C53	1.447 (2)
C11—H11	0.9500	C53—H53	0.9500
C12—C21	1.532 (2)	C54—C55	1.533 (2)
C12—C13	1.534 (2)	C54—C59	1.534 (2)
C12—C18	1.540 (2)	C54—C63	1.538 (2)
C13—C14	1.531 (2)	C55—C56	1.544 (2)
C13—H13A	0.9900	C55—H55A	0.9900
C13—H13B	0.9900	C55—H55B	0.9900
C14—C17	1.527 (3)	C56—C57	1.530 (3)
C14—C15	1.529 (3)	C56—C60	1.534 (3)
C14—H14	1.0000	C56—H56	1.0000
C15—C16	1.536 (3)	C57—C58	1.534 (2)
C15—H15A	0.9900	C57—H57A	0.9900
C15—H15B	0.9900	C57—H57B	0.9900
C16—C19	1.524 (3)	C58—C62	1.531 (3)
C16—C18	1.541 (2)	C58—C59	1.538 (2)
C16—H16	1.0000	C58—H58	1.0000
C17—C20	1.530 (3)	C59—H59A	0.9900
C17—H17A	0.9900	C59—H59B	0.9900
C17—H17B	0.9900	C60—C61	1.530 (2)
C18—H18A	0.9900	C60—H60A	0.9900
C18—H18B	0.9900	C60—H60B	0.9900
C19—C20	1.533 (3)	C61—C63	1.533 (2)
C19—H19A	0.9900	C61—C62	1.534 (3)
C19—H19B	0.9900	C61—H61	1.0000
C20—C21	1.547 (2)	C62—H62A	0.9900
C20—H20	1.0000	C62—H62B	0.9900
C21—H21A	0.9900	C63—H63A	0.9900
C21—H21B	0.9900	C63—H63B	0.9900
C22—C31	1.414 (2)	C64—C73	1.415 (2)
C22—C23	1.439 (2)	C64—C65	1.438 (2)
C23—C24	1.356 (3)	C65—C66	1.357 (3)
C23—H23	0.9500	C65—H65	0.9500
C24—C25	1.424 (3)	C66—C67	1.418 (3)
C24—H24	0.9500	C66—H66	0.9500
C25—C26	1.415 (2)	C67—C68	1.416 (2)
C25—C30	1.426 (2)	C67—C72	1.423 (2)
C26—C27	1.367 (3)	C68—C69	1.360 (3)
C26—H26	0.9500	C68—H68	0.9500
C27—C28	1.404 (3)	C69—C70	1.398 (3)
C27—H27	0.9500	C69—H69	0.9500
C28—C29	1.377 (2)	C70—C71	1.377 (3)
C28—H28	0.9500	C70—H70	0.9500
C29—C30	1.417 (2)	C71—C72	1.410 (3)
C29—H29	0.9500	C71—H71	0.9500
C30—C31	1.454 (2)	C72—C73	1.459 (2)
C31—C32	1.443 (2)	C73—C74	1.436 (2)
C32—H32	0.9500	C74—H74	0.9500
C33—C42	1.532 (2)	C75—C80	1.533 (2)
C33—C34	1.536 (2)	C75—C84	1.538 (2)
C33—C38	1.538 (2)	C75—C76	1.539 (2)
C34—C35	1.532 (2)	C76—C77	1.536 (2)
C34—H34A	0.9900	C76—H76A	0.9900
C34—H34B	0.9900	C76—H76B	0.9900
C35—C39	1.532 (3)	C77—C78	1.531 (2)
C35—C36	1.533 (3)	C77—C83	1.534 (2)
C35—H35	1.0000	C77—H77	1.0000
C36—C37	1.531 (3)	C78—C79	1.532 (3)
C36—H36A	0.9900	C78—H78A	0.9900
C36—H36B	0.9900	C78—H78B	0.9900
C37—C40	1.529 (3)	C79—C81	1.531 (3)
C37—C38	1.539 (2)	C79—C80	1.533 (2)
C37—H37	1.0000	C79—H79	1.0000
C38—H38A	0.9900	C80—H80A	0.9900
C38—H38B	0.9900	C80—H80B	0.9900
C39—C41	1.528 (3)	C81—C82	1.533 (3)
C39—H39A	0.9900	C81—H81A	0.9900
C39—H39B	0.9900	C81—H81B	0.9900
C40—C41	1.531 (3)	C82—C83	1.530 (3)
C40—H40A	0.9900	C82—C84	1.543 (2)
C40—H40B	0.9900	C82—H82	1.0000
C41—C42	1.539 (2)	C83—H83A	0.9900
C41—H41A	1.0000	C83—H83B	0.9900
C42—H42A	0.9900	C84—H84A	0.9900
C42—H42B	0.9900	C84—H84B	0.9900
			
O1—Co1—O2	115.23 (5)	O3—Co2—O4	114.40 (5)
O1—Co1—N1	94.93 (5)	O3—Co2—N3	94.94 (5)
O2—Co1—N1	117.15 (5)	O3—Co2—N4	116.97 (5)
O1—Co1—N2	115.95 (5)	O4—Co2—N3	115.20 (5)
O2—Co1—N2	93.16 (5)	O4—Co2—N4	94.63 (5)
N1—Co1—N2	122.20 (6)	N3—Co2—N4	122.25 (6)
C22—O2—Co1	121.77 (11)	C43—O3—Co2	123.99 (10)
C1—O1—Co1	126.36 (11)	C64—O4—Co2	125.92 (11)
C11—N1—C12	119.94 (14)	C53—N3—C54	119.82 (13)
C11—N1—Co1	121.19 (11)	C53—N3—Co2	120.68 (11)
C12—N1—Co1	118.86 (10)	C54—N3—Co2	119.41 (10)
C32—N2—C33	120.42 (14)	C74—N4—C75	116.28 (13)
C32—N2—Co1	119.07 (11)	C74—N4—Co2	121.06 (11)
C33—N2—Co1	120.51 (10)	C75—N4—Co2	122.59 (10)
O1—C1—C10	124.93 (15)	O3—C43—C52	125.09 (15)
O1—C1—C2	116.18 (15)	O3—C43—C44	116.04 (15)
C10—C1—C2	118.88 (15)	C52—C43—C44	118.87 (15)
C3—C2—C1	121.71 (16)	C45—C44—C43	121.92 (16)
C3—C2—H2	119.1	C45—C44—H44	119.0
C1—C2—H2	119.1	C43—C44—H44	119.0
C2—C3—C4	121.53 (16)	C44—C45—C46	121.48 (16)
C2—C3—H3	119.2	C44—C45—H45	119.3
C4—C3—H3	119.2	C46—C45—H45	119.3
C5—C4—C9	120.22 (16)	C47—C46—C51	120.43 (15)
C5—C4—C3	120.82 (16)	C47—C46—C45	120.80 (15)
C9—C4—C3	118.94 (15)	C51—C46—C45	118.76 (15)
C6—C5—C4	121.44 (16)	C48—C47—C46	121.45 (16)
C6—C5—H5	119.3	C48—C47—H47	119.3
C4—C5—H5	119.3	C46—C47—H47	119.3
C5—C6—C7	119.04 (16)	C47—C48—C49	118.71 (16)
C5—C6—H6	120.5	C47—C48—H48	120.6
C7—C6—H6	120.5	C49—C48—H48	120.6
C8—C7—C6	120.70 (17)	C50—C49—C48	121.30 (17)
C8—C7—H7	119.7	C50—C49—H49	119.4
C6—C7—H7	119.7	C48—C49—H49	119.4
C7—C8—C9	122.00 (16)	C49—C50—C51	121.70 (16)
C7—C8—H8	119.0	C49—C50—H50	119.2
C9—C8—H8	119.0	C51—C50—H50	119.2
C8—C9—C4	116.53 (15)	C50—C51—C46	116.39 (15)
C8—C9—C10	123.89 (15)	C50—C51—C52	123.94 (15)
C4—C9—C10	119.55 (15)	C46—C51—C52	119.66 (15)
C1—C10—C11	122.76 (15)	C43—C52—C53	122.64 (15)
C1—C10—C9	119.27 (15)	C43—C52—C51	119.27 (15)
C11—C10—C9	117.87 (15)	C53—C52—C51	118.01 (15)
N1—C11—C10	128.12 (16)	N3—C53—C52	127.69 (15)
N1—C11—H11	115.9	N3—C53—H53	116.2
C10—C11—H11	115.9	C52—C53—H53	116.2
N1—C12—C21	115.87 (13)	N3—C54—C55	115.18 (13)
N1—C12—C13	105.69 (13)	N3—C54—C59	107.40 (13)
C21—C12—C13	109.11 (14)	C55—C54—C59	108.96 (14)
N1—C12—C18	108.36 (13)	N3—C54—C63	107.59 (13)
C21—C12—C18	108.13 (14)	C55—C54—C63	108.29 (13)
C13—C12—C18	109.56 (14)	C59—C54—C63	109.32 (13)
C14—C13—C12	110.64 (14)	C54—C55—C56	109.89 (13)
C14—C13—H13A	109.5	C54—C55—H55A	109.7
C12—C13—H13A	109.5	C56—C55—H55A	109.7
C14—C13—H13B	109.5	C54—C55—H55B	109.7
C12—C13—H13B	109.5	C56—C55—H55B	109.7
H13A—C13—H13B	108.1	H55A—C55—H55B	108.2
C17—C14—C15	110.40 (16)	C57—C56—C60	110.09 (15)
C17—C14—C13	108.84 (15)	C57—C56—C55	109.65 (14)
C15—C14—C13	108.88 (15)	C60—C56—C55	109.34 (14)
C17—C14—H14	109.6	C57—C56—H56	109.2
C15—C14—H14	109.6	C60—C56—H56	109.2
C13—C14—H14	109.6	C55—C56—H56	109.2
C14—C15—C16	109.54 (15)	C56—C57—C58	109.03 (14)
C14—C15—H15A	109.8	C56—C57—H57A	109.9
C16—C15—H15A	109.8	C58—C57—H57A	109.9
C14—C15—H15B	109.8	C56—C57—H57B	109.9
C16—C15—H15B	109.8	C58—C57—H57B	109.9
H15A—C15—H15B	108.2	H57A—C57—H57B	108.3
C19—C16—C15	109.78 (16)	C62—C58—C57	109.56 (15)
C19—C16—C18	108.89 (15)	C62—C58—C59	109.35 (15)
C15—C16—C18	109.59 (15)	C57—C58—C59	109.30 (14)
C19—C16—H16	109.5	C62—C58—H58	109.5
C15—C16—H16	109.5	C57—C58—H58	109.5
C18—C16—H16	109.5	C59—C58—H58	109.5
C14—C17—C20	109.55 (15)	C54—C59—C58	110.28 (14)
C14—C17—H17A	109.8	C54—C59—H59A	109.6
C20—C17—H17A	109.8	C58—C59—H59A	109.6
C14—C17—H17B	109.8	C54—C59—H59B	109.6
C20—C17—H17B	109.8	C58—C59—H59B	109.6
H17A—C17—H17B	108.2	H59A—C59—H59B	108.1
C12—C18—C16	109.87 (14)	C61—C60—C56	109.07 (14)
C12—C18—H18A	109.7	C61—C60—H60A	109.9
C16—C18—H18A	109.7	C56—C60—H60A	109.9
C12—C18—H18B	109.7	C61—C60—H60B	109.9
C16—C18—H18B	109.7	C56—C60—H60B	109.9
H18A—C18—H18B	108.2	H60A—C60—H60B	108.3
C16—C19—C20	109.60 (15)	C60—C61—C63	109.44 (14)
C16—C19—H19A	109.7	C60—C61—C62	109.29 (15)
C20—C19—H19A	109.7	C63—C61—C62	109.75 (14)
C16—C19—H19B	109.7	C60—C61—H61	109.5
C20—C19—H19B	109.7	C63—C61—H61	109.5
H19A—C19—H19B	108.2	C62—C61—H61	109.5
C17—C20—C19	109.64 (16)	C58—C62—C61	109.67 (14)
C17—C20—C21	109.43 (16)	C58—C62—H62A	109.7
C19—C20—C21	109.43 (15)	C61—C62—H62A	109.7
C17—C20—H20	109.4	C58—C62—H62B	109.7
C19—C20—H20	109.4	C61—C62—H62B	109.7
C21—C20—H20	109.4	H62A—C62—H62B	108.2
C12—C21—C20	109.54 (14)	C61—C63—C54	110.17 (14)
C12—C21—H21A	109.8	C61—C63—H63A	109.6
C20—C21—H21A	109.8	C54—C63—H63A	109.6
C12—C21—H21B	109.8	C61—C63—H63B	109.6
C20—C21—H21B	109.8	C54—C63—H63B	109.6
H21A—C21—H21B	108.2	H63A—C63—H63B	108.1
O2—C22—C31	124.78 (15)	O4—C64—C73	124.69 (15)
O2—C22—C23	116.39 (15)	O4—C64—C65	116.66 (16)
C31—C22—C23	118.81 (16)	C73—C64—C65	118.63 (16)
C24—C23—C22	121.34 (17)	C66—C65—C64	121.26 (18)
C24—C23—H23	119.3	C66—C65—H65	119.4
C22—C23—H23	119.3	C64—C65—H65	119.4
C23—C24—C25	121.70 (17)	C65—C66—C67	122.16 (17)
C23—C24—H24	119.1	C65—C66—H66	118.9
C25—C24—H24	119.1	C67—C66—H66	118.9
C26—C25—C24	120.98 (16)	C68—C67—C66	121.41 (17)
C26—C25—C30	119.98 (17)	C68—C67—C72	119.76 (18)
C24—C25—C30	119.04 (16)	C66—C67—C72	118.79 (16)
C27—C26—C25	121.35 (17)	C69—C68—C67	121.75 (19)
C27—C26—H26	119.3	C69—C68—H68	119.1
C25—C26—H26	119.3	C67—C68—H68	119.1
C26—C27—C28	119.25 (17)	C68—C69—C70	118.81 (18)
C26—C27—H27	120.4	C68—C69—H69	120.6
C28—C27—H27	120.4	C70—C69—H69	120.6
C29—C28—C27	120.68 (18)	C71—C70—C69	121.1 (2)
C29—C28—H28	119.7	C71—C70—H70	119.5
C27—C28—H28	119.7	C69—C70—H70	119.5
C28—C29—C30	121.78 (17)	C70—C71—C72	121.64 (19)
C28—C29—H29	119.1	C70—C71—H71	119.2
C30—C29—H29	119.1	C72—C71—H71	119.2
C29—C30—C25	116.92 (16)	C71—C72—C67	116.92 (16)
C29—C30—C31	123.84 (16)	C71—C72—C73	123.71 (16)
C25—C30—C31	119.23 (16)	C67—C72—C73	119.34 (16)
C22—C31—C32	121.74 (15)	C64—C73—C74	122.65 (15)
C22—C31—C30	119.71 (15)	C64—C73—C72	119.65 (15)
C32—C31—C30	118.09 (15)	C74—C73—C72	117.69 (15)
N2—C32—C31	127.25 (16)	N4—C74—C73	128.48 (16)
N2—C32—H32	116.4	N4—C74—H74	115.8
C31—C32—H32	116.4	C73—C74—H74	115.8
N2—C33—C42	115.05 (13)	N4—C75—C80	108.12 (13)
N2—C33—C34	107.43 (13)	N4—C75—C84	112.62 (13)
C42—C33—C34	108.71 (14)	C80—C75—C84	108.37 (13)
N2—C33—C38	107.47 (13)	N4—C75—C76	109.25 (13)
C42—C33—C38	108.98 (14)	C80—C75—C76	108.52 (13)
C34—C33—C38	109.06 (13)	C84—C75—C76	109.87 (14)
C35—C34—C33	110.33 (14)	C77—C76—C75	110.22 (13)
C35—C34—H34A	109.6	C77—C76—H76A	109.6
C33—C34—H34A	109.6	C75—C76—H76A	109.6
C35—C34—H34B	109.6	C77—C76—H76B	109.6
C33—C34—H34B	109.6	C75—C76—H76B	109.6
H34A—C34—H34B	108.1	H76A—C76—H76B	108.1
C39—C35—C34	109.61 (15)	C78—C77—C83	109.92 (14)
C39—C35—C36	109.33 (16)	C78—C77—C76	108.90 (14)
C34—C35—C36	109.37 (15)	C83—C77—C76	109.11 (14)
C39—C35—H35	109.5	C78—C77—H77	109.6
C34—C35—H35	109.5	C83—C77—H77	109.6
C36—C35—H35	109.5	C76—C77—H77	109.6
C37—C36—C35	109.23 (14)	C77—C78—C79	109.62 (14)
C37—C36—H36A	109.8	C77—C78—H78A	109.7
C35—C36—H36A	109.8	C79—C78—H78A	109.7
C37—C36—H36B	109.8	C77—C78—H78B	109.7
C35—C36—H36B	109.8	C79—C78—H78B	109.7
H36A—C36—H36B	108.3	H78A—C78—H78B	108.2
C40—C37—C36	109.98 (15)	C81—C79—C78	109.46 (15)
C40—C37—C38	109.15 (15)	C81—C79—C80	109.03 (14)
C36—C37—C38	109.70 (15)	C78—C79—C80	109.51 (14)
C40—C37—H37	109.3	C81—C79—H79	109.6
C36—C37—H37	109.3	C78—C79—H79	109.6
C38—C37—H37	109.3	C80—C79—H79	109.6
C33—C38—C37	109.75 (14)	C75—C80—C79	110.57 (14)
C33—C38—H38A	109.7	C75—C80—H80A	109.5
C37—C38—H38A	109.7	C79—C80—H80A	109.5
C33—C38—H38B	109.7	C75—C80—H80B	109.5
C37—C38—H38B	109.7	C79—C80—H80B	109.5
H38A—C38—H38B	108.2	H80A—C80—H80B	108.1
C41—C39—C35	109.38 (15)	C79—C81—C82	109.61 (14)
C41—C39—H39A	109.8	C79—C81—H81A	109.7
C35—C39—H39A	109.8	C82—C81—H81A	109.7
C41—C39—H39B	109.8	C79—C81—H81B	109.7
C35—C39—H39B	109.8	C82—C81—H81B	109.7
H39A—C39—H39B	108.2	H81A—C81—H81B	108.2
C37—C40—C41	109.28 (15)	C83—C82—C81	109.61 (15)
C37—C40—H40A	109.8	C83—C82—C84	109.54 (15)
C41—C40—H40A	109.8	C81—C82—C84	109.31 (15)
C37—C40—H40B	109.8	C83—C82—H82	109.5
C41—C40—H40B	109.8	C81—C82—H82	109.5
H40A—C40—H40B	108.3	C84—C82—H82	109.5
C39—C41—C40	109.48 (15)	C82—C83—C77	109.77 (14)
C39—C41—C42	109.80 (16)	C82—C83—H83A	109.7
C40—C41—C42	109.43 (15)	C77—C83—H83A	109.7
C39—C41—H41A	109.4	C82—C83—H83B	109.7
C40—C41—H41A	109.4	C77—C83—H83B	109.7
C42—C41—H41A	109.4	H83A—C83—H83B	108.2
C33—C42—C41	109.89 (14)	C75—C84—C82	109.50 (14)
C33—C42—H42A	109.7	C75—C84—H84A	109.8
C41—C42—H42A	109.7	C82—C84—H84A	109.8
C33—C42—H42B	109.7	C75—C84—H84B	109.8
C41—C42—H42B	109.7	C82—C84—H84B	109.8
H42A—C42—H42B	108.2	H84A—C84—H84B	108.2
